# Proximity labeling reveals a BIN2 signaling network

**DOI:** 10.1093/plcell/koad006

**Published:** 2023-01-18

**Authors:** Leiyun Yang

**Affiliations:** Assistant Features Editor, The Plant Cell, American Society of Plant Biologists, USA; Department of Plant Pathology, College of Plant Protection, Nanjing Agricultural University, Key Laboratory of Integrated Management of Crop Diseases and Pests, Ministry of Education, Nanjing 210095, China; The Key Laboratory of Plant Immunity, Nanjing Agricultural University, Nanjing 210095, China

Kinases are enzymes that add phosphate groups to substrate proteins, thereby altering the cellular functions of substrates. Glycogen synthase kinase 3 (GSK3) is a major cellular signal transduction hub, acts in diverse signaling pathways, and phosphorylates over a hundred cellular proteins in eukaryotes. BIN2, the best-characterized GSK3-like kinase in plants, regulates various developmental and physiological processes including brassinosteroid signaling, cellular development, and stress responses (Li et al., 2021). Given its many important duties as a hub for diverse signaling pathways, there has long been interest in identifying a more comprehensive repertoire of BIN2 substrates. However, the interactions between enzymes and substrates tend to be dynamic and transient, and cannot easily be captured by classical techniques like co-immunoprecipitation (co-IP), which requires stable interactions.

In new work, Tae-Wuk Kim and colleagues (Kim et al., 2022) used the combinatorial approach of TurboID-based proximity labeling and mass spectrometry (TbPL-MS) to improve the resolution of BIN2 substrates. Mapping the BIN2 signaling network based on their approach revealed intriguing crosstalk between BIN2 substrates and the protein modification *O*-linked β-*N*-acetylglucosamine (O-GlcNAc; see Figure).

**Figure:**
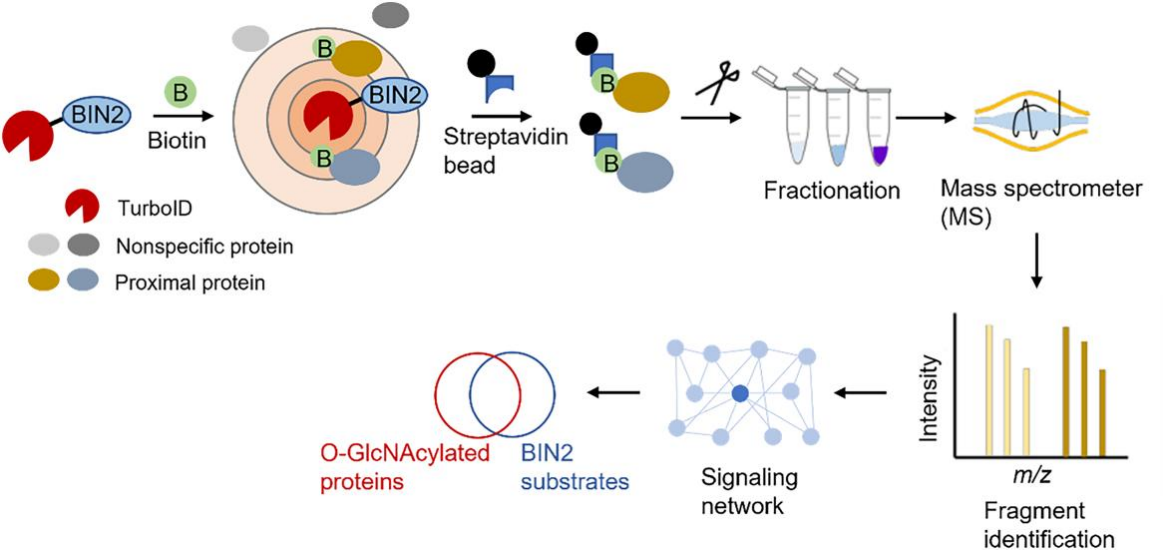
Simplified schematic diagram of the workflow of TbPL-MS followed by BIN2 signaling network analyses. The TurboID fused with BIN2 labels BIN2-proximal proteins that are pulled down by streptavidin beads and processed through fractionation, MS, protein identification, and signaling network mapping. Adapted from Kim et al. (2022), Figures 1A and 3A.

TurboID is a highly active biotin ligase used for proximity-based labeling of nearby proteins in the presence of biotin. Streptavidin beads, which specifically bind to biotin, are used to pull down biotin-labeled proteins, which can then be detected by immunoblotting or identified by mass spectrometry (see Figure). The authors compared the sensitivity and specificity of TbPL with traditional co-IP via the known interaction between BIN2 and its substrate, BZR1. Via immunoblotting, they found that BZR1 was pulled down 10 times more using TbPL than co-IP relative to a yellow fluorescent protein (YFP) control, illustrating that TbPL is a sensitive and highly specific alternative to low-throughput co-IP.

Using transgenic plants expressing TbID fused to either BIN2 or a YFP control, the group performed TbPL-MS to quantitatively distinguish BIN2-proximal proteins from any non-specific proteins tagged by YFP. This remarkably led to the identification of 482 BIN2-proximal proteins. These BIN2-proximal proteins include known BIN2 substrates and many unknown interactors, especially components of the brassinosteroid signaling pathway. To distinguish BIN2 substrates among these proximal proteins, the authors treated BIN2 transgenic plants with bikinin, a BIN2 inhibitor, and performed quantitative phosphoproteomic analysis. Of the BIN2-proximal proteins, 169 (41.2%) showed dephosphorylation after bikinin treatment, and 12 randomly selected were also validated as BIN2 substrates in a kinase assay, indicating most of them are BIN2 substrates. The newly identified BIN2 substrates are involved in several key cellular functions, including transcriptional and translational regulation, vesicular trafficking, cell wall integrity, and phototropic responses. These data broaden our understanding of the scope of the BIN2 regulatory network to include even more key cellular processes.

O-GlcNAc is another protein modification critical for regulating cellular homeostasis in animals, as well as growth and development in plants (Hart, 2019; Sun, 2021). Interestingly, the authors found that ∼46% of O-GlcNAc-modified proteins in plants were either proximal to BIN2, showed BIN2-dependent phosphorylation, or both. Additionally, about 22% of the putative GSK3 substrates were both phosphorylated and O-GlcNAcylated in mice. Thus, their study has indirectly revealed extensive crosstalk between GSK3 and O-GlcNAcylation-mediated signaling pathways in eukaryotes.

This study further establishes the robustness of TbPL-MS in capturing transient and dynamic protein–protein interactions in plants. The BIN2-proximal proteins and the network analyses showcased in this study provide a much deeper understanding of GSK family proteins in eukaryotes. It would be interesting to explore how BIN2 functions specifically in various signaling pathways and how BIN2-mediated phosphorylation interacts with O-GlcNAcylation.
